# Quantifying upright positioning accuracy with optical surface tracking in radiotherapy

**DOI:** 10.1002/acm2.70527

**Published:** 2026-03-19

**Authors:** Yusuke Nomura, Sodai Tanaka, Hideyuki Takei, Kinji Maeda, Takuro Takekoshi, Hideki Iwakami, Hirotoshi Takiyama, Minoru Tajiri, Shunsuke Yonai, Hideyuki Mizuno, Yoshiyuki Iwata, Taku Inaniwa, Hitoshi Ishikawa

**Affiliations:** ^1^ Department of Accelerator and Medical Physics Institute for Quantum Medical Science National Institutes for Quantum Science and Technology Chiba Japan; ^2^ Radiological Technology Section Department of Medical Technology QST Hospital National Institutes for Quantum Science and Technology Chiba Japan; ^3^ QST Hospital National Institutes for Quantum Science and Technology Chiba Japan

**Keywords:** inter‐fractional, intra‐fractional, setup accuracy, upright radiotherapy

## Abstract

**Purpose:**

This study quantified inter‐ and intra‐fractional setup accuracies in the upright posture and compared them among setups with different immobilization methods.

**Methods:**

Two and four setups were examined for abdominal and head and neck (HN) cancer treatments, respectively. Fifteen asymptomatic volunteers were positioned to a replicated chair of an upright radiotherapy platform with leg immobilization devices, backrest attachments, thermoplastic masks, and vacuum cushions. The 3D positions of the subject body and masks were monitored by calculating 3D point clouds of 26 surface markers from three camera images. The inter‐fractional setup errors were calculated by repeating the same setup five times. The intra‐fractional displacements were evaluated while the subjects remained in the setups for 20 min. These setup errors and displacements were compared among the setups with different immobilization methods. The intra‐fractional displacements were also compared between this study and previous studies in the supine posture.

**Results:**

Inter‐fractional setup errors in the abdominal setups were reduced from 6.6 ± 3.3 to 3.9 ± 1.7 mm by using the masks. The HN setup using both the leg immobilization devices and backrest attachments had the setup errors of 2.9 ± 1.7 mm. This was smaller than the setup errors observed in three other HN setups that did not use either or both of the devices together. Intra‐fractional displacements of these abdominal and HN setups with the immobilization devices were 1.9 ± 1.1 and 1.8 ± 1.5 mm, respectively, which were smaller than those in the other setups. These displacements were equivalent to those in the previous studies.

**Conclusions:**

Utilizing the masks increased upright setup accuracy in the abdominal setup. The leg immobilization devices and backrest attachments provided the highest setup accuracy in the upright HN setup. These findings will be useful to expand the applicability of upright radiotherapy for various cancer treatments.

## 1 INTRODUCTION

Radiotherapy in the upright posture is a treatment technique in which fixed horizontal beams are delivered to patients who sit or stand on a rotating chair. Numerous potential advantages of the upright positioning for radiotherapy have been discussed,^1^, ^2^, ^3^ in comparison to conventional radiotherapy in the supine or prone posture. For instance, the upright positioning is useful to decrease respiratory motions while keeping strong correlations between the internal organ motions and surrogate signals.^1^, ^2^ The radiotherapy platforms in the upright posture also become much less expensive and smaller in size, and they have simpler management than the conventional supine platforms.^3^ This is advantageous especially in charged particle therapies such as proton therapy or carbon ion radiotherapy (CIRT)^4^, ^5^, ^6^, ^7^ because a rotating gantry, which has a length of over 10 m in the CIRT platforms and contains many complicated beam control devices,^8^, ^9^ is not required to deliver beams to patients from 360° beam angles.

Despite these promising benefits, the upright radiotherapy has clinically been conducted for only limited cancers such as eye or head and neck (HN) cancer.^4^, ^5^, ^6^, ^7^, ^10^, ^11^ To expand its applicability for various other types of cancers, target‐specific immobilization methods need to be developed. However, many previous studies^4^, ^5^, ^6^, ^12^, ^13^ investigated only one upright setup at the head regions. Therefore, it is still unclear which immobilization device or setup is useful for precise upright positioning, and the applicability for other cancers has not been revealed. Moreover, these previous studies did not monitor the intra‐fractional motions during treatment. To evaluate the effects of immobilization devices for each specific target accurately, both the inter‐fractional setup errors and real‐time intra‐fractional motions need to be compared among multiple possible setup methods.

One effective technique to assess the setup accuracy is optical surface imaging.^14^, ^15^, ^16^, ^17^ This technique provides 3D positions of markers or a patient's body surface. Although it cannot measure internal anatomy like portal imaging or computed tomography, it is noninvasive and easier to implement, and it provides 3D body surface motions in nearly real time. While it has commonly been performed for initial patient setup or motion monitoring in conventional supine radiotherapy,^16^, ^17^, ^18^, ^19^, ^20^, ^21^ Boisbouvier et al.^22^, ^23^ demonstrated its use for setup accuracy assessment at pelvic and breast regions in the upright posture.

This study aims to quantify the inter‐ and intra‐fractional setup accuracies of two abdominal and four HN upright setups and compare them to evaluate the effects of immobilization methods on upright positioning. Based on the marker‐based surface imaging technique,^16^, ^17^ the inter‐ and intra‐fractional motions were measured with high‐resolution optical cameras and 3D surface imaging software. These setup accuracies were compared between the setups to quantitatively evaluate the effects of the immobilization devices on the setup accuracies. Moreover, the intra‐fractional setup accuracy was compared between those in this study and previous studies in the supine posture. While the experiment and immobilization setups were based on clinical CIRT treatments at National Institutes for Quantum Science and Technology (QST), the findings of this study can also be applied for any other beam types of radiotherapy. To the best of the authors’ knowledge, this is the first study to quantitatively assess the setup accuracy of multiple setups for abdominal and HN treatments in upright posture.

## 2 METHODS

### 2.1 Experimental setup

**FIGURE 1:**
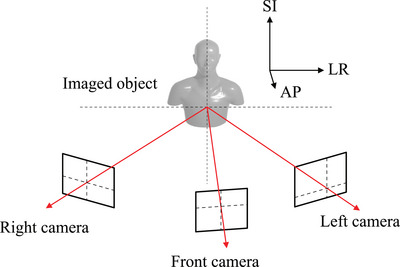
Schematic diagram of the experimental setup. Red arrows represent optical light beams emitted from the surface of an imaged object, which were detected by three optical cameras.

The experimental setup is shown in Figure 1. A demonstration chair which replicates the clinical upright radiotherapy system EVE (Leo Cancer Care, Crawley, UK) was utilized. This demonstration chair had the same design and functions as the EVE except that it was not able to translate and rotate. It had five adjustable immobilization components as discussed by Boisbouvier et al.^22^: a backrest angle, a seat pan angle, seat translation along the superior‐inferior (SI) direction, shin rest translation along the anterior‐posterior (AP) direction, and heel stop translation along the AP direction. Multiple index points were regularly placed at the lateral side of the backrest to attach an arm rest at subject‐specific positions. Three sets of a CMOS camera (exo183CGE, SVS‐Vistek GmbH, Gilching, Germany) and an optical lens with a focal length of 25 mm (C11‐2520‐12M, Basler AG, Ahrensburg, Germany) were used to measure subject 3D body positions. The f‐number of each lens was set to four in order to get a depth‐of‐field value greater than 50 cm and to put the imaged objects into focus. The acquired images were 5496 × 3672 pixels in size.

To replicate clinical treatment rooms, horizontal and vertical laser lines were projected by three laser generators (SK14P, Makita Corporation, Aichi, Japan) positioned at the front, left, and right sides of the chair. The height of the horizontal lasers was set at 115 cm above the floor. The vertical lasers from the front and the lateral laser generators represented the left‐right (LR) and AP coordinate planes of the chair, respectively, and these laser lines intersected at a vertical line which was 15 cm anterior to the backrest, corresponding to the vertical rotation axis of EVE.

### 2.2 Subject enrollment

Fifteen asymptomatic male volunteers were enrolled as subjects, all of whom provided informed written consent. Their average age, height, and weight were 45.3 years old (range: 28–72 years old), 172.4 cm (160–183 cm), and 72.4 kg (60–96 kg), respectively. Prior to the experiment, a radiation oncologist assured their health status and confirmed their eligibility for this study. This study was approved by the Institutional Review Board of QST and registered in the University Hospital Medical Information Network Clinical Trials Registry (UMIN ID: UMIN000055769).

### 2.3 Setup methods

**FIGURE 2:**
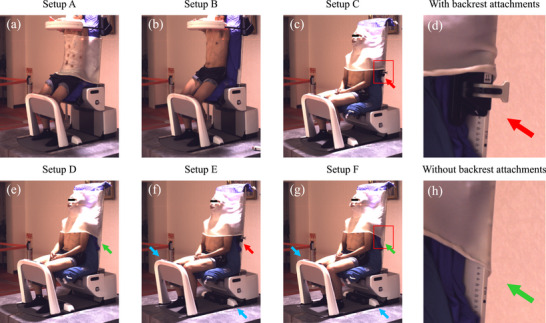
Left camera images of a representative subject in six setups. (a) and (b) show abdominal setups A and B, and (c), (e), (f), and (g) illustrate HN setups C to F. (d) and (h) are cropped images at areas shown by red rectangles in (c) and (g), respectively. Thermoplastic masks were supported with backrest attachments only in the setups C and E (as pointed out with red arrows), while those in the setups D and F were not (green arrows). The shin rest and heel stop in the setups E and F were not attached to the subject (blue arrows).

Six subject setups, denoted as setups A–F, were investigated as shown in Figure 2 and Table 1. The backrest angle was set to 5° backward, and the back and hip of the subjects were fixed with vacuum cushions (BlueBAG, 3C‐Medical Intelligence GmbH, Landsberg am Lech, Germany and Klarity Vacuum Bags, Klarity Medical Products, Heath, OH, USA) in each of the setups.

**TABLE 1:** Comparison of immobilization setups. The shin rest, heel stop, mask, and backrest attachments were used in the setups with check marks (✓).

Setup	Body region	Positions of horizontal laser line	Initial alignment	Shin rest and heel stop	Mask	Backrest attachments
A	Abdomen	Axilla	Lateral index of mask	✓	✓	
B			Lasers and skin marks	✓		
C	Head and neck	External auditory meatus	Lateral index of mask	✓	✓	✓
D				✓	✓	
E					✓	✓
F					✓	

#### 2.3.1 Abdominal setups

Setups A and B were the setups for abdominal or pelvic cancer treatment. Both setups used the arm rest, shin rest, and heel stop, but only setup A used a thermoplastic mask (Shell Fitter, Kuraray Trading Co., Ltd., Tokyo, Japan). A laser‐based alignment was performed around the subject's axilla in setup B. This alignment position was higher than the abdominal region, as Boisbouvier et al.^23^ previously reported that aligning the upper abdominal region was more challenging than aligning the lower abdominal region in the upright posture.

#### 2.3.2 HN setups

Setups C, D, E, and F simulated the setups for HN cancer treatment. The horizontal laser lines were located at a height comparable to that of the external auditory meatuses. The shin rest and heel stop were not used in setups E and F (as marked by blue arrows in Figure 2). Backrest attachments (ONE Respiratory Belt, Civco, Orange City, IA, USA) were used to support the masks only in setups C and E (red arrows).

### 2.4 Image data acquisition

**FIGURE 3:**
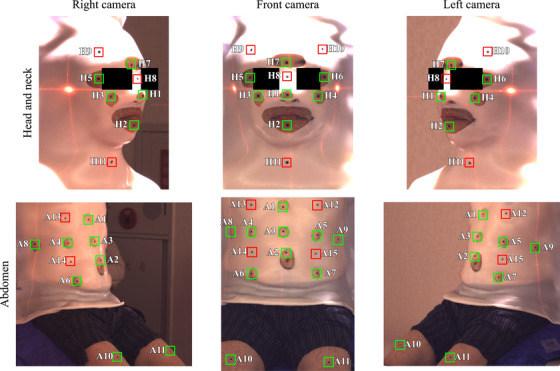
Positions of mask holes and markers in abdominal and HN setups. Green and red squares represent regions of the body and mask markers, respectively. Detailed marker positions are described in Table S1.

The experiment was conducted for each subject over two days. On the first day, the subject‐specific thermoplastic masks and vacuum cushions were prepared by experienced radiation therapists. The chair components were first adjusted to align the subject body, and these positions were recorded. Subsequently, the vacuum cushions were molded to support the subject's hip and back, and the masks were then made. After hardening the masks, holes around the eyes, nostrils, mouth, and other openings were made in the masks as shown in Figure 3. Moreover, the lateral index points on the backrest were marked on the masks to reproduce the mask setups accurately. Two vacuum cushions and one mask were made for either the abdominal or HN setup, and they were used for the setups of the same body region.

On the second day, the intra‐ and inter‐fractional motions were measured. The experiment workflow is illustrated in Figure S2. First, 26 black circle markers (diameter, 5 mm) were put on the body surface and the masks (Figure 3). After aligning the subject with the vacuum cushions and chair components at the positions recorded on the first day, the laser lines were marked on the body surface in setup B. In the other setups, the subject was aligned by setting the masks based on the marked lateral index points. The intra‐fractional motion measurement was then performed by consecutively acquiring the camera images with a time interval of 5 s while the subject remained aligned for 20 min. The first image frame was defined as the reference image. Subsequently, the inter‐fractional motions were measured by repeating the alignment and single image acquisition for five times within the same day. The subject alignment was performed with the marked laser lines in setup B and with the lateral index points in the other setups.

The experiment was conducted for 15 and 9 subjects in the abdominal and HN setups, respectively. The cameras were controlled with the image acquisition library Harvester version 1.4.3,^24^ and the median time lag of image acquisition among three cameras was 5 ms.

### 2.5 Inter‐ and intra‐fractional motion calculations

To calculate 3D positions of the body and masks accurately, intrinsic and extrinsic camera parameters needed to be well calibrated for each camera. The intrinsic camera parameters were first calibrated with Zhang's method,^25^ and the extrinsic camera parameters were then calibrated using a 41 × 58 grid plate with a grid space of 5.0 mm (Figure S1). The gird plate images at six predefined positions were acquired, and pixel positions of the grid points in these images were manually detected. The extrinsic camera parameters were calibrated by minimizing a reprojection error between the detected pixel positions and those projected from the 3D grid plate positions. These calibrated parameters were used for further image processing and analysis.

The inter‐ and intra‐fractional displacements were calculated using the following four steps.

**FIGURE 4:**
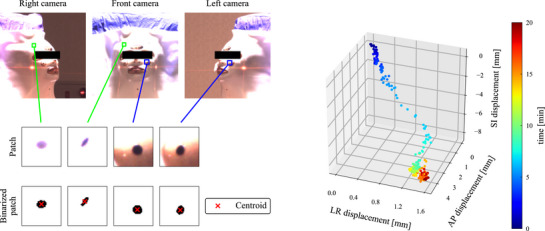
Marker detection process for the triangulation (left) and a calculated motion trajectory of a representative marker (right). Green and blue boxes represented patch positions of the same mask and body markers, respectively. The centroids of the black (i.e. zero value) pixels in the binarized patches are marked as the 2D marker positions in the image coordinate system with red cross marks. The 3D scatter data indicate the 3D positions of the body marker during the intra‐fractional motion measurement.

1. The marker positions in the acquired images were detected by calculating the centroids of pixels with zero pixel value in the binarized patches (Figure 4). These positions were manually reviewed and corrected if necessary.
2. Triangulation was performed for these markers to obtain 3D point clouds of the body and mask markers. A pair of the front and left camera images were mainly used to calculate the left side of the markers, while the front and right camera images were used for the other side. The left and right camera images were used only when the front camera could not capture the markers clearly.
3. A rigid point cloud registration was applied with an iterative closest point (ICP) algorithm^26^ to calculate a body transformation matrix *M*_body_ and mask transformation matrix *M*_mask_. Source and target point clouds in the ICP registration were set to the markers in the reference and other images, respectively. The origin of transformation matrices in the abdominal and HN setups was set at the same SI position of A2 and H1 markers in the reference images along the vertical rotation axis of EVE, respectively. One challenge was that the ICP registration could not be directly applied for the A1–A9 markers because they moved non‐rigidly due to respiration. To alleviate this issue, these marker positions in the images acquired during the intra‐fractional motion measurement were smoothed over five subsequent frames prior to applying the ICP registration.^27^ The A10 and A11 markers were analyzed individually to assess thigh position stability.
4. Translational and rotational displacements of the body and masks along the LR, AP, and SI directions were derived from *M*_body_ and *M*_mask_, respectively. The rotational displacements were represented by pitch (rotation around the LR axis), roll (AP axis), and yaw (SI axis).

The camera calibration, triangulation, and ICP registration were performed with in‐house software using the computer vision libraries, OpenCV version 4.10.0^28^ and Open3D version 0.19.0.^29^

### 2.6 Data analysis and comparison

Accuracy of the surface imaging system was verified by calculating grid position errors along the LR, AP, and SI directions, as well as 3D position errors. The grid plate (Figure S1) was placed at another predefined position that was not used for the camera calibration, and the ground truth grid positions were obtained. The 3D grid positions were calculated by applying the triangulation with both the front‐left, front‐right, and LR camera pairs, and these errors against the ground truth positions were evaluated. The 3D position errors were calculated from an L2‐norm of the grid position errors along each 3D axis.

The inter‐ and intra‐fractional setup accuracies were evaluated and compared among the setups. The inter‐fractional setup accuracy was evaluated by calculating an overall setup error *μ*, systematic setup error Σ, and random setup error *σ*.^30^ *μ* and Σ were defined as the mean and standard deviation of individual mean setup errors over all subjects, while *σ* was the mean of the individual random setup errors over all subjects. The inter‐fractional 3D setup errors were also compared between these setups. The intra‐fractional setup accuracy was evaluated by calculating the translational and rotational displacements as a function of time. Moreover, a relative rigid transformation matrix $M_{\mathrm{rel}}$ was calculated to assess the relative body displacements from the masks in the following equation.

(1) $$M_{\mathrm{rel}}=M_{\mathrm{body}}\cdot M_{\mathrm{mask}}^{-1}$$

where $M_{\mathrm{mask}}^{-1}$ denotes the inverse mask transformation matrix.

The intra‐fractional setup accuracy was further evaluated by comparing the time period in which all translational and rotational displacements were within tolerance thresholds during the measurement of 20 min, which was referred to as beam delivery time (BDT).^18^, ^19^, ^20^, ^21^ The six time periods in which the displacement on each translational or rotational axis was within the respective threshold were also compared. Based on previous studies,^18^, ^21^ the tolerance thresholds were set to ± 2 mm/ ± 1° and ± 1.5 mm/ ± 1° for the abdominal and HN setups, respectively. Moreover, the intra‐fractional displacements in this study were compared with those in previous studies^18^, ^19^, ^20^, ^21^ which measured the body surface motions of actual patients in supine posture.

Statistical differences in means and variances between the abdominal setups were evaluated with Welch's *t*‐test and Brown‐Forsythe test, respectively. For multiple comparisons among the HN setups, these tests were applied for all pairs of the HN setups, and *p* values were adjusted with the Holm–Bonferroni method. All statistical analyses were applied with a significance level of 0.05 by using the scientific libraries, Scipy version 1.17.0^31^ and Statsmodels version 0.14.4.^32^

## 3 RESULTS

### 3.1 Calculation accuracy of 3D surface imaging system

Table 2 lists the grid position errors along the LR, AP, and SI directions, as well as the 3D position errors. Mean absolute and 95th percentile errors were less than or equal to 0.32 mm and 0.59 mm, respectively. The 3D position errors were not greater than 0.45 mm. Therefore, these systems had sufficiently high calculation accuracy of the 3D marker positions for the setup accuracy assessment.

**TABLE 2:** Calculation accuracy of the 3D surface imaging system. Mean and standard deviation (SD) values of absolute grid position errors calculated from the front‐left, front‐right, and left‐right camera pairs are shown.

		LR	AP	SI	3D
Front‐left cameras	Mean ± SD [mm]	0.23 ± 0.16	0.17 ± 0.10	0.32 ± 0.06	0.45 ± 0.13
	95th percentile [mm]	0.53	0.36	0.41	0.70
Front‐right cameras	Mean ± SD [mm]	0.23 ± 0.16	0.22 ± 0.12	0.24 ± 0.10	0.42 ± 0.18
	95th percentile [mm]	0.53	0.40	0.38	0.71
Left‐right cameras	Mean ± SD [mm]	0.27 ± 0.18	0.18 ± 0.10	0.28 ± 0.07	0.45 ± 0.16
	95th percentile [mm]	0.59	0.33	0.36	0.72

Abbreviation: LR = left‐right, AP = anterior‐posterior, SI = superior‐inferior.

### 3.2 Inter‐fractional setup errors

**FIGURE 5:**
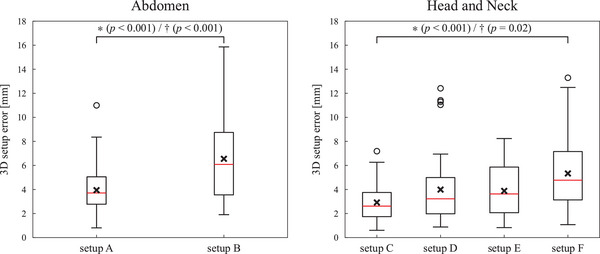
Comparison of the inter‐fractional 3D setup errors for the abdominal and HN setups. Asterisk (∗) and dagger (†) represent a statistically significant difference in means and variances (*p* < 0.05), respectively. *p* values for the HN setups were adjusted with the Holm‐Bonferroni method. Cross marks represent mean values.

The inter‐fractional setup errors of the abdominal and HN setups are shown in Table 3. The 3D setup errors are also compared in Figure 5. The *μ* values were within ± 1.9 mm and ± 0.8° in all setups. For the abdominal setups, setup A provided an equivalent or smaller Σ and σ values compared to setup B. The mean and standard deviation values of 3D setup errors significantly decreased from 6.6 ± 3.3 mm for setup B to 3.9 ± 1.7 mm for setup A (*p* < 0.001 for both Welch's *t*‐test and the Brown‐Forsythe test). For the HN setups, setup C had equivalent or smaller setup errors than the other HN setups. In particular, setup C had significantly smaller setup errors than setup F (adjusted *p* < 0.001 for Welch's *t*‐test and adjusted *p* = 0.02 for the Brown‐Forsythe test). The 3D setup errors were 2.9 ± 1.7, 4.0 ± 2.8, 3.9 ± 2.1, and 5.3 ± 3.1 mm in setups C, D, E, and F, respectively.

**TABLE 3:** Comparison of the inter‐fractional setup errors in the abdominal and HN setups.

	Translation [mm]												Rotation [°]								
	LR			AP			SI			3D			Pitch			Roll			Yaw		
Setup	*μ*	Σ	*σ*	*μ*	Σ	*σ*	*μ*	Σ	*σ*	*μ*	Σ	*σ*	*μ*	Σ	*σ*	*μ*	Σ	*σ*	*μ*	Σ	*σ*
A	0.6	1.4	1.3	0.3	1.7	1.6	1.6	2.2	1.4	3.9	1.3	1.2	−0.5	0.8	0.4	0.1	0.8	0.5	0.1	0.5	0.5
B	0.4	2.1	1.6	0.8	4.2	2.7	1.9	3.6	2.8	6.6	2.2	2.6	−0.8	1.7	0.9	−0.2	0.6	0.3	0.7	0.9	0.7
C	0.0	0.7	0.6	−0.9	1.7	1.0	0.5	2.4	1.0	2.9	1.5	0.9	−0.1	0.7	0.4	−0.4	0.9	0.5	0.3	0.7	0.5
D	1.7	3.2	1.4	0.0	1.5	0.9	1.2	1.6	1.7	4.0	2.5	1.4	0.1	1.3	0.5	0.3	1.2	0.7	0.4	1.0	0.6
E	0.3	1.4	0.5	−0.3	1.8	1.0	0.9	3.3	1.7	3.9	1.8	1.1	0.2	0.6	0.4	−0.5	0.5	0.6	0.5	0.8	0.5
F	1.5	3.7	1.0	0.2	2.0	0.6	0.2	4.3	1.3	5.3	3.0	1.2	0.5	0.9	0.4	0.3	0.6	0.5	0.1	0.7	0.5

Abbreviation: LR = left‐right, AP = anterior‐posterior, SI = superior‐inferior. μ = overall setup error, Σ = overall systematic setup error, σ = overall random setup error.

### 3.3 Intra‐fractional displacements

**FIGURE 6:**
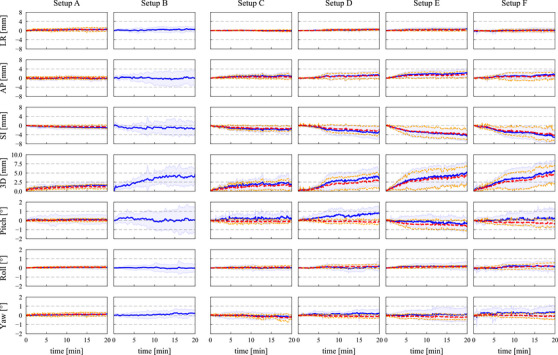
Comparison of the intra‐fractional displacements. Blue solid lines and bars represent mean and standard deviation values of the body displacements over all subjects. Red and orange dashed lines depict mean and standard deviation values of the mask displacements. These displacements along the left‐right (LR), anterior‐posterior (AP), and superior‐inferior (SI) directions became positive when the body and masks moved toward left, anterior, and superior directions relative to those in the reference images, respectively.

**FIGURE 7:**
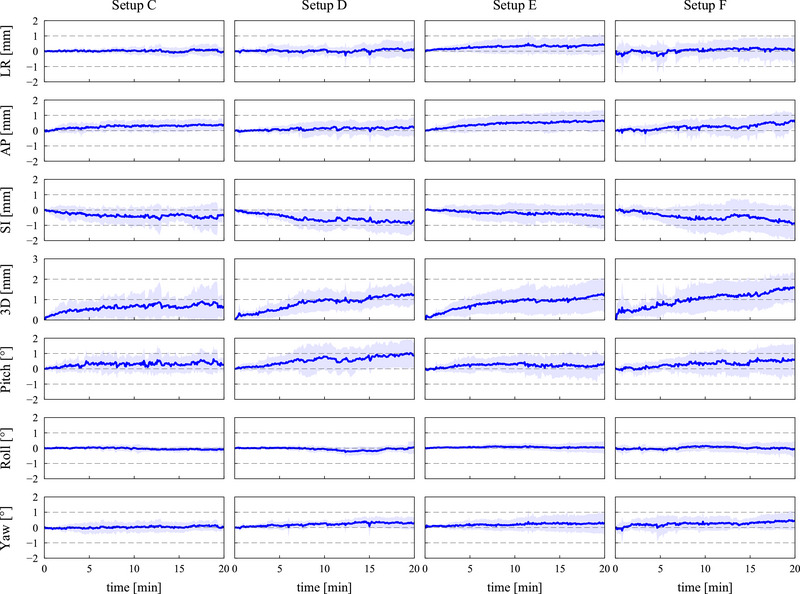
Comparison of the intra‐fractional displacements of the subject body relative to the masks in the HN setups. Solid lines and bars represent mean and standard deviation values of the relative body displacements against the masks for all subjects.

The intra‐fractional displacements of the abdominal and HN setups for all subject body and masks are shown in Figure 6. The intra‐fractional displacements of individual markers are also illustrated in Figures S3 and S4. Comparisons of the intra‐fractional displacements at 20 min are shown in Figure S5. Moreover, the intra‐fractional body displacements relative to the masks in the HN setups are shown in Figure 7. Among the markers, 4% of them could not be detected because they were removed or covered by the masks, subject's clothing, or subject's abdominal fat during the measurement.

The intra‐fractional displacements at the abdominal region were more stable in setup A than setup B. Their standard deviations at 20 min were significantly reduced from 3.8 and 3.0 mm for setup B to 1.4 and 0.7 mm for setup A along the AP (*p* = 0.009) and SI (*p* = 0.001) directions, respectively. The standard deviations of pitch and roll displacements in setup A were also smaller than those in setup B (*p* = 0.008 and 0.006, respectively). The mean and standard deviation values of 3D displacements at 20 min were reduced from 4.6 ± 1.9 mm for setup B to 1.9 ± 1.1 mm for setup A (*p* < 0.001 for Welch's *t*‐test and *p* = 0.04 for the Brown‐Forsythe test). On the other hand, differences of the mean translational and rotational displacements were within ± 0.8 mm and ± 0.9° over all time periods. The mean displacements at 20 min had no significant differences between these setups (*p* > 0.05). The A10 and A11 markers were also stable, and their translational displacements were less than 0.6 mm over all time periods (Figure S3).

The intra‐fractional displacements at the HN region became large for the first 5 min in most of the setups. The anterior and inferior translational displacements were observed for both the body and masks in all setups. The 3D displacements at 20 min were 1.8 ± 1.5, 3.4 ± 2.1, 5.1 ± 2.7, and 5.5 ± 3.0 mm in setups C, D, E, and F, respectively. Setup C provided significantly smaller displacements than setups E and F along the SI (adjusted *p* = 0.03 for both comparisons) and 3D (adjusted *p* = 0.04) directions. Moreover, the intra‐fractional body displacements relative to the masks (Figure 7) show that the relative displacements were within ± 2.0 mm and smaller especially for the AP and SI directions than the intra‐fractional body displacements (Figure 6). No significant differences were found among the relative displacements at 20 min in these setups (adjusted *p* > 0.05).

### 3.4 BDT

**FIGURE 8:**
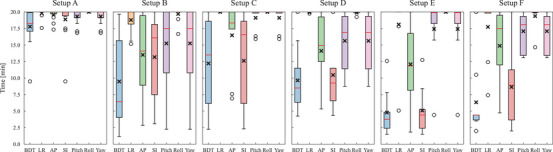
Comparison of the BDT and time periods with tolerable displacement for each translational or rotational axis. Cross and circle marks represent mean values and outliers, respectively.

The BDT and time periods in which the displacement on each translational or rotational axis was within the tolerance threshold are illustrated in Figure 8. For the abdominal setups with the threshold of ± 2 mm/ ± 1°, the mean and standard deviation of the BDT values for all subjects were 17.8 ± 2.7 and 9.5 ± 6.6 min in setups A and B, respectively. Setup A provided significantly longer time than setup B (*p* < 0.001). For the HN setups with the threshold of ± 1.5 mm/ ± 1°, the BDT was 12.2 ± 7.1, 9.6 ± 5.2, 4.8 ± 3.5, and 6.3 ± 5.6 min in setups C, D, E, and F, respectively. No statistically significant differences were observed among these setups (adjusted *p* > 0.05).

## 4 DISCUSSION

This study quantified and compared the inter‐ and intra‐fractional setup accuracies in the abdominal and HN setups in the upright posture. The abdominal setup with the masks (setup A) provided smaller intra‐fractional 3D displacements than that without the masks (setup B) (Figure S5). The variances in the translational and rotational displacements were also smaller for setup A than setup B. On the other hand, the mean displacements were equivalent between these setups (Figure 6). These results indicate that, although the masks did not reduce overall intra‐fractional displacements, the respiratory motions and subject‐specific motions were effectively suppressed with the masks. Since the masks also reduced the inter‐factional 3D setup errors (Figure 5) and increased the BDT (Figure 8), the masks were considered a crucial immobilization device. In the HN setups, setup C using the shin rest, heel stop, and backrest attachments provided equivalent or higher setup accuracy than the other HN setups. Comparison between setups C and E and between setups D and F show that the shin rest and heel stop effectively decreased the intra‐fractional displacements by preventing the subjects from gradually drifting especially along the SI direction (Figure 6). Moreover, comparison between setups C and D and between setups E and F revealed that the subjects moved together with the masks and the backrest attachments did not effectively support the masks (Figure 7). On the other hand, setups C and E had over 1.0 mm smaller inter‐fractional 3D setup errors than setups D and F, respectively, mainly because the reproducibility of mask setup increased with the assistance of the backrest attachments. Therefore, utilizing the backrest attachments was an effective technique for the initial alignment, and the intra‐fractional motions are expected to be further reduced when the masks are directly attached with the backrest.^4^, ^5^, ^6^, ^12^, ^13^

Comparison of the intra‐fractional body surface displacements between this study and previous studies^18^, ^19^, ^20^, ^21^ was made using the summarized results in Table 4. Since this study focused on the setup accuracy regardless of the direction of displacements, the mean displacements are represented as absolute values. The AP displacement in setup A was remarkably smaller than that in two previous studies^18^, ^20^ possibly due to reduced respiratory motions with the masks. For the HN setups, all displacements at 3 min in setup C were within ± 1.0 mm and ± 0.5° of those in the previous studies,^19^, ^21^ indicating clinically consistent setup accuracy. These results indicated that setups A and C have potential to provide consistent setup accuracy for clinical treatments.

**TABLE 4:** Comparison of the intra‐fractional body surface displacements between this study and previous studies^18^, ^19^, ^20^, ^21^ (mean ± standard deviation). The mean motions are represented as absolute values.

				Translation [mm]				Rotation [°]		
	Posture	Body part	Time [min]	LR	AP	SI	3D	Pitch	Roll	Yaw
* Heinzerling et al.^18^	Supine	Thorax or abdomen	NA	1.00	1.35	1.96	3.32	0.376	0.286	0.202
Apicella et al.^20^	Supine	Pelvic	7–8	0.07	1.20	0.95	—	—	—	—
			14.9	0.26	1.55	1.00	—	—	—	—
**This study**	Upright	Abdomen (setup A)	3	0.3 ± 0.4	0.2 ± 0.8	0.5 ± 0.9	1.1 ± 0.8	0.1 ± 0.4	0.0 ± 0.1	0.0 ± 0.1
			5	0.3 ± 0.5	0.2 ± 0.8	0.5 ± 0.6	1.2 ± 0.5	0.1 ± 0.4	0.0 ± 0.1	0.0 ± 0.2
			10	0.4 ± 0.7	0.0 ± 0.9	0.7 ± 0.7	1.4 ± 0.6	0.1 ± 0.5	0.1 ± 0.1	0.1 ± 0.2
			15	0.4 ± 0.8	0.0 ± 0.9	0.9 ± 0.9	1.6 ± 0.7	0.1 ± 0.5	0.1 ± 0.2	0.1 ± 0.2
			20	0.7 ± 1.0	0.1 ± 1.4	1.0 ± 0.7	1.9 ± 1.1	0.1 ± 0.4	0.1 ± 0.2	0.2 ± 0.3
Zhao et al.^21^	Supine	HN	NA	0.07 ± 0.92	0.11 ± 0.89	0.10 ± 0.83	—	0.02 ± 0.41	0.02 ± 0.44	0.08 ± 0.43
Han et al.^19^	Supine	HN (vacuum fixation)	2.94	0.02 ± 0.26	0.01 ± 0.18	0.06 ± 0.30	—	0.01 ± 0.13	0.02 ± 0.19	0.01 ± 0.13
		HN (open‐face mask)	2.78	0.01 ± 0.40	0.06 ± 0.20	0.02 ± 0.35	—	0.00 ± 0.16	0.05 ± 0.23	0.02 ± 0.21
**This study**	Upright	HN (setup C)	3	0.1 ± 0.3	0.3 ± 0.6	0.6 ± 0.8	1.0 ± 0.8	0.1 ± 0.3	0.0 ± 0.1	0.0 ± 0.3
			5	0.1 ± 0.3	0.6 ± 0.8	1.0 ± 1.0	1.5 ± 0.8	0.2 ± 0.4	0.0 ± 0.2	0.0 ± 0.3
			10	0.1 ± 0.2	1.0 ± 0.8	1.7 ± 1.1	2.0 ± 1.3	0.2 ± 0.4	0.1 ± 0.3	0.1 ± 0.4
			15	0.1 ± 0.4	0.6 ± 0.9	1.4 ± 1.1	1.7 ± 1.2	0.2 ± 0.4	0.0 ± 0.3	0.1 ± 0.3
			20	0.1 ± 0.5	0.8 ± 1.1	1.5 ± 1.2	1.8 ± 1.5	0.2 ± 0.4	0.0 ± 0.2	0.2 ± 0.3

^*^Heinzerling et al.^18^ reported only the cases in which the displacements were greater than their tolerance thresholds (±2mm / ±1° for 2 s).

Abbreviation: LR = left‐right, AP = anterior‐posterior, SI = superior‐inferior, NA = not available.

This study has three main differences compared to the studies of Boisbouvier et al.^22^, ^23^ who have also investigated the setup accuracy of EVE with the surface imaging technique. First, this study calculated the 3D marker positions by using the triangulation with submillimeter accuracy (Table 2), while the two previous studies used an optical guidance and tracking system (OGTS) which supported visual comparison and manual alignment between two images. Because the OGTS only provided the 2D translational shifts of an acquired image relative to the reference image, Boisbouvier et al. regarded these shifts in the front and lateral camera images as the 3D motions without considering rotational motions nor making a quantitative accuracy evaluation. Second, this study considered the HN and abdominal setups, while Boisbouvier et al. focused on pelvic and breast setups. Third, this study used different immobilization devices and setups based on the CIRT in contrast to two previous studies for photon radiotherapy. Therefore, this study offers novel insights for the upright positioning.

There are several limitations to the present study. The setup accuracies in this study might be different from those of clinical radiotherapy with the clinically representative EVE. The intra‐fractional motions due to the chair rotation could not be assessed because the chair used in this study was a demonstration prototype and cannot rotate. Moreover, the inter‐fractional setup accuracy might be different from that of clinical radiotherapy because the experiments were performed for the asymptomatic subjects within a single day. Another major limitation is that correlations between the body surface motions and internal anatomy could not be assessed. Although several studies^17^, ^33^ have shown these correlations in the supine posture, only very limited studies have investigated them in the upright posture.^2^ Because of these limitations, the inter‐fractional setup errors in this study could not directly be compared with those in previous studies. In addition to these major limitations, the setup accuracies of female subjects could not be assessed because only male volunteers applied to this study. A few body marker positions differed between the reference and the other images because the markers were removed and reattached in the middle of the experiments. However, this difference is considered not serious because the ICP registrations were performed with all body markers. Moreover, calculation error of the camera system due to thermal effects was not quantitatively evaluated. Similar to previous studies,^34^, ^35^ uncertainty on the order of sub‐millimeters might be included in this study. Further investigations will be made to solve these issues in the future.

The chair system and demonstrated setups have various issues to be addressed for clinical applications. Some subjects perceived pain in, or numbness of, their shins, hips, or arms when using the arm rest. Moreover, making the HN masks in the upright posture was more difficult especially at the nose and jaw than making them in the supine posture because they sagged down toward the inferior direction due to gravity. Therefore, the quality of the HN masks was dependent on the expertise of the radiation therapists making them. Further improvements in the chair and immobilization devices are required for more precise and comfortable treatment in the upright posture.

## 5 CONCLUSION

The inter‐ and intra‐fractional setup accuracies in the upright posture were quantified and compared for abdominal and HN regions. The setup accuracy in the abdominal setup increased with application of the masks. The shin rest and heel stop prevented the subject's head from inferiorly drifting in the HN setups. The intra‐fractional displacements in this study were consistent with those in previous studies. Although further investigations of the correlations between the surface motions and internal anatomy are warranted, the findings in this study provide several novel insights and evidence toward clinical applications.

## AUTHOR CONTRIBUTIONS

*Project design development*: Taku Inaniwa, Hideki Iwakami, Kinji Maeda, Yusuke Nomura, Hideyuki Takei, Takuro Takekoshi, and Sodai Tanaka. *Data acquisition*: Hideki Iwakami, Kinji Maeda, Yusuke Nomura, Hideyuki Takei, Takuro Takekoshi, Sodai Tanaka, and Hirotoshi Takiyama. *Data analysis*: Yusuke Nomura. *Manuscript writing*: Yusuke Nomura. *Supervision*: Taku Inaniwa, Hitoshi Ishikawa, Yoshiyuki Iwata, Hideyuki Mizuno, Minoru Tajiri, and Shunsuke Yonai.

## CONFLICT OF INTEREST STATEMENT

The Leo Cancer Care lent the demonstration chair and camera system to QST without charge. Yusuke Nomura also received travel funding and support for attending research meetings without any honoraria from Leo Cancer Care. All other authors have no other relevant conflicts of interests to disclose.

## Supporting information



Supporting Information

## Data Availability

Data are not available for sharing due to an institutional review board restriction.
