# Supplementary material for: Quantifying upright positioning accuracy with optical surface tracking in radiotherapy
Source: J Appl Clin Med Phys. 2026 Mar 19;27(3):e70527. doi: 10.1002/acm2.70527 (PMC13093279; doi:10.1002/acm2.70527)
Supplement: Supplementary file 1 — Supporting Information [file ACM2-27-e70527-s001.pdf]

Supplementary Material: Quantifying upright positioning accuracy with optical surface tracking in radiotherapy

Table S1. Description of body and mask marker positions for the abdominal and head and neck setups.

Abdominal markers

ID		Position
A1		Xiphoid process on midsagittal line
A2		Above umbilicus on midsagittal line
A3		Center point between markers A1 and A2
A4		Right side of marker A3
A5		Left side of marker A3
A6	Body	Under the marker A4 at height of right iliac crest
A7		Under the marker A5 at height of left iliac crest
A8		Right side of marker A4
A9		Left side of marker A5
A10		Right thigh
A11		Left thigh
A12		Above the marker A5 and left side of marker A1
A13	Mask	Above the marker A4 and right side of marker A1
A14		Under the marker A4 and right side of marker A2
A15		Under the marker A5 and left side of marker A2

Head and neck markers

ID		Position
H1		Tip of nose
H2		Under lower vermillion border
H3		Right zygomatic process
H4	Body	Left zygomatic process
H5		Right side of right lateral canthus
H6		Left side of left lateral canthus
H7		Central glabella
H8		Center point between markers H1 and H7
H9	Mask	Right upper head
H10		Left upper head
H11		Central throat

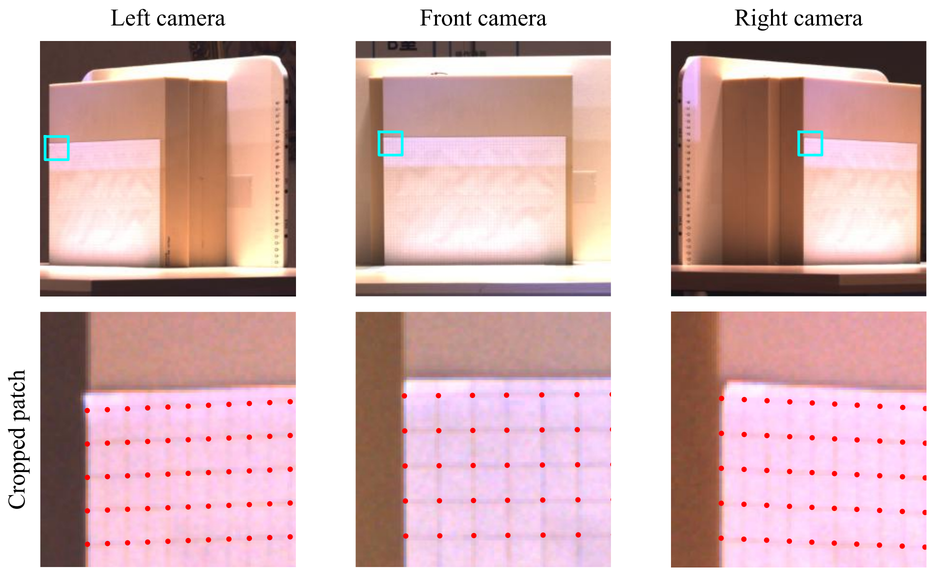

Figure S1. Grid plate images for camera calibration and verification. The bottom images show the cropped images at areas shown by cyan boxes in the upper images. Red dots represent detected grid positions.

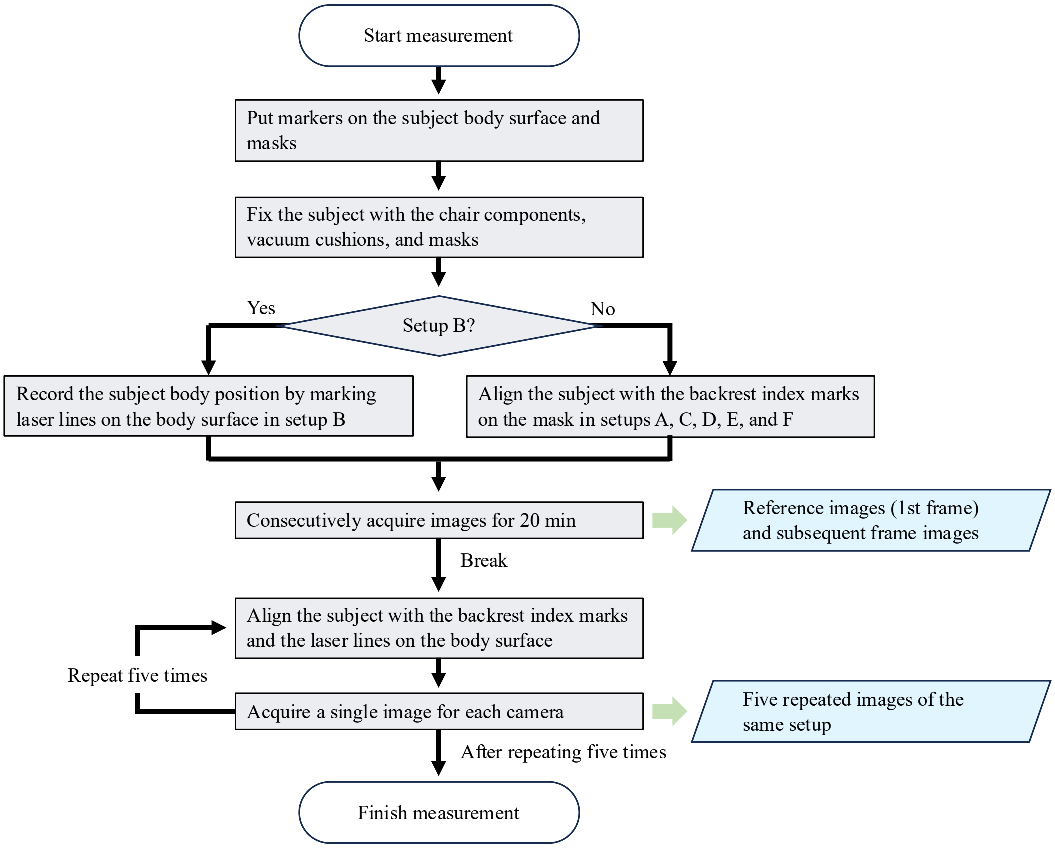

Figure S2. Diagram of experiment workflow. Gray rectangles and diamond-shaped boxes represent steps of the experiment, while blue parallelograms represent acquired image data.

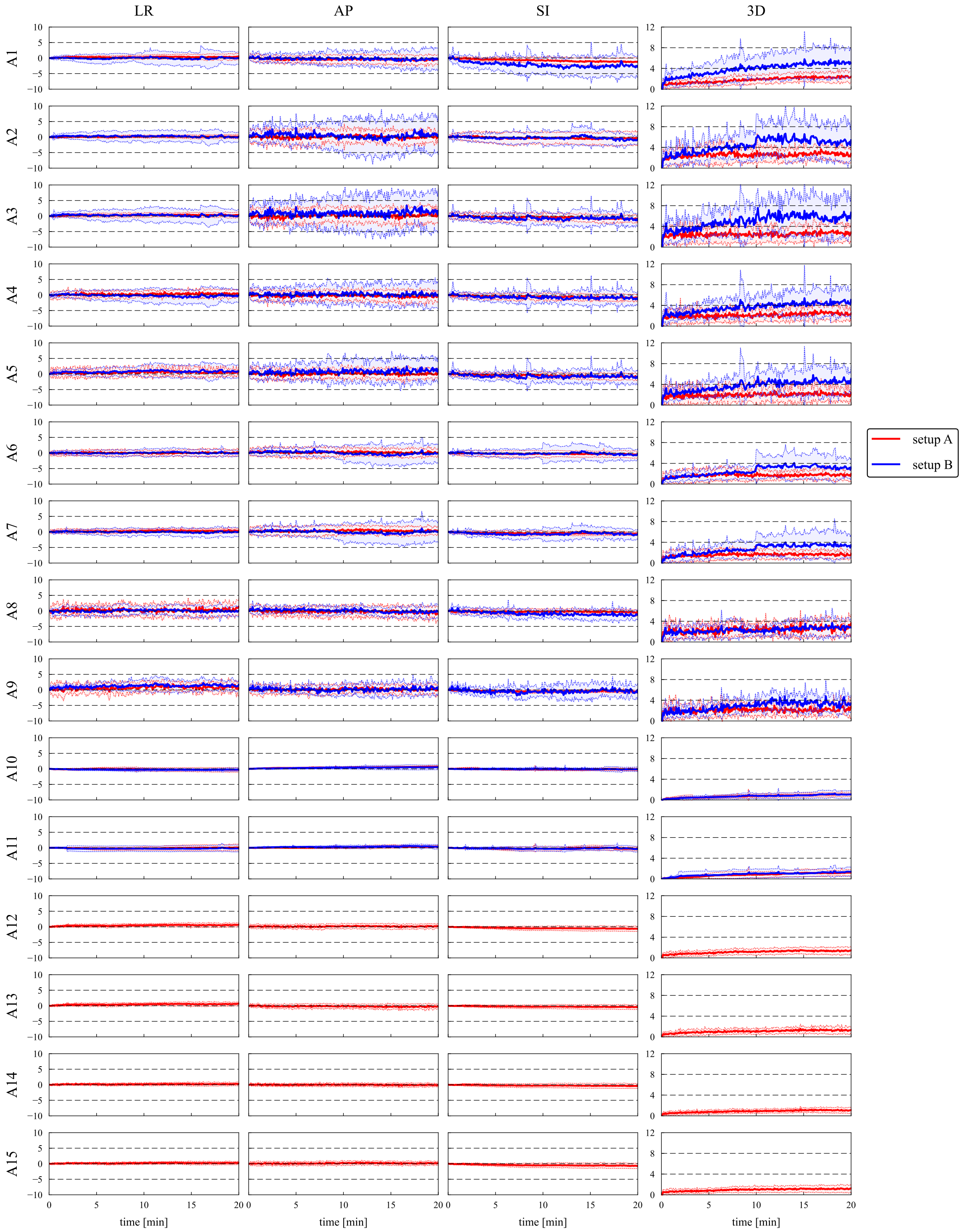

Figure S3. The intra-fractional displacements [mm] of the body and mask markers in setups A and B. Numbers on the left side denote the identification numbers of markers as shown in Table S1. Lines and shaded bars respectively represent mean and standard deviation values over all subjects.

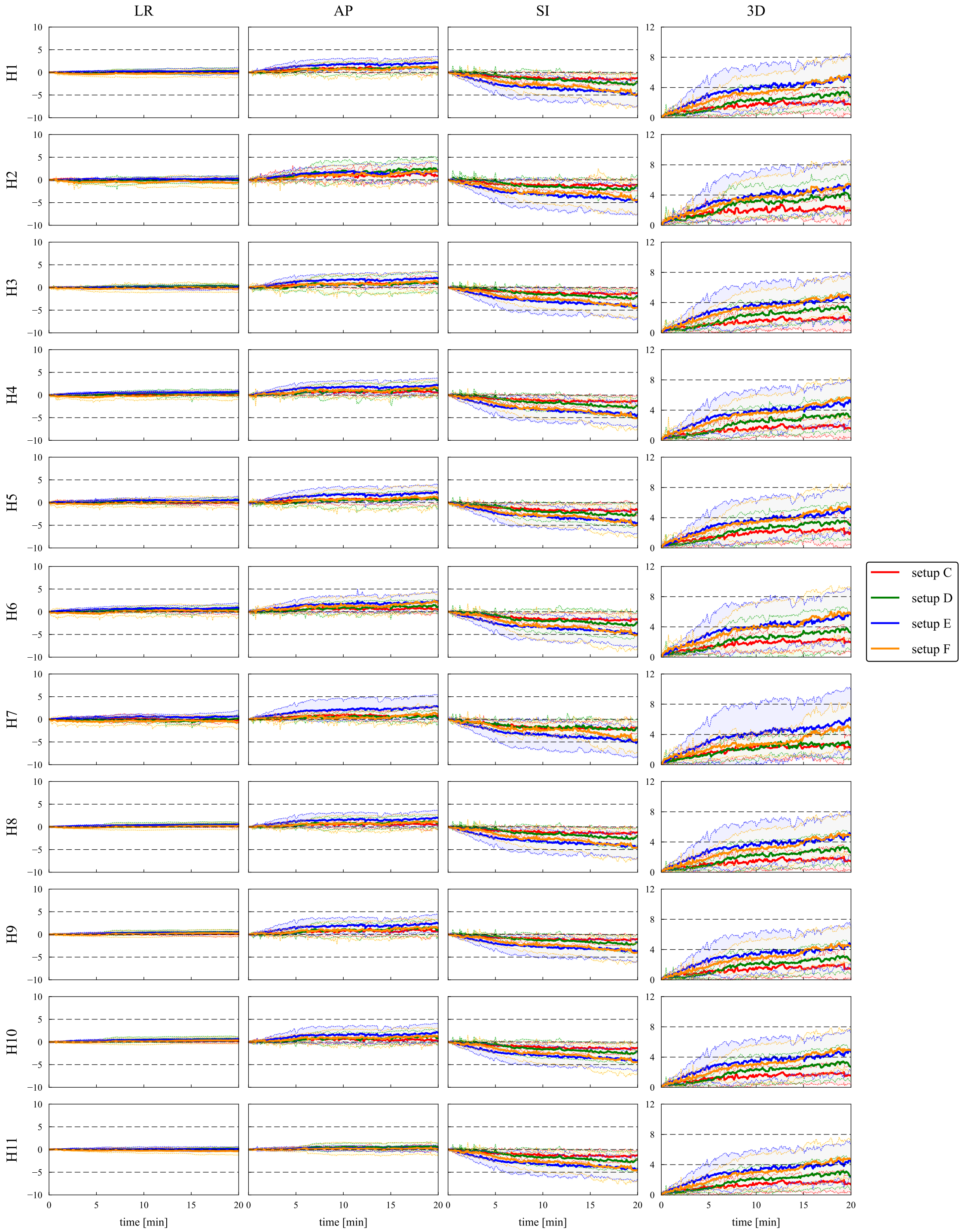

Figure S4. The intra-fractional displacements [mm] of the body and mask markers in setups C, D, E, and F. Numbers on the left side denote the identification numbers of markers as shown in Table S1. Lines and shaded bars respectively represent average and standard deviation values over all subjects.

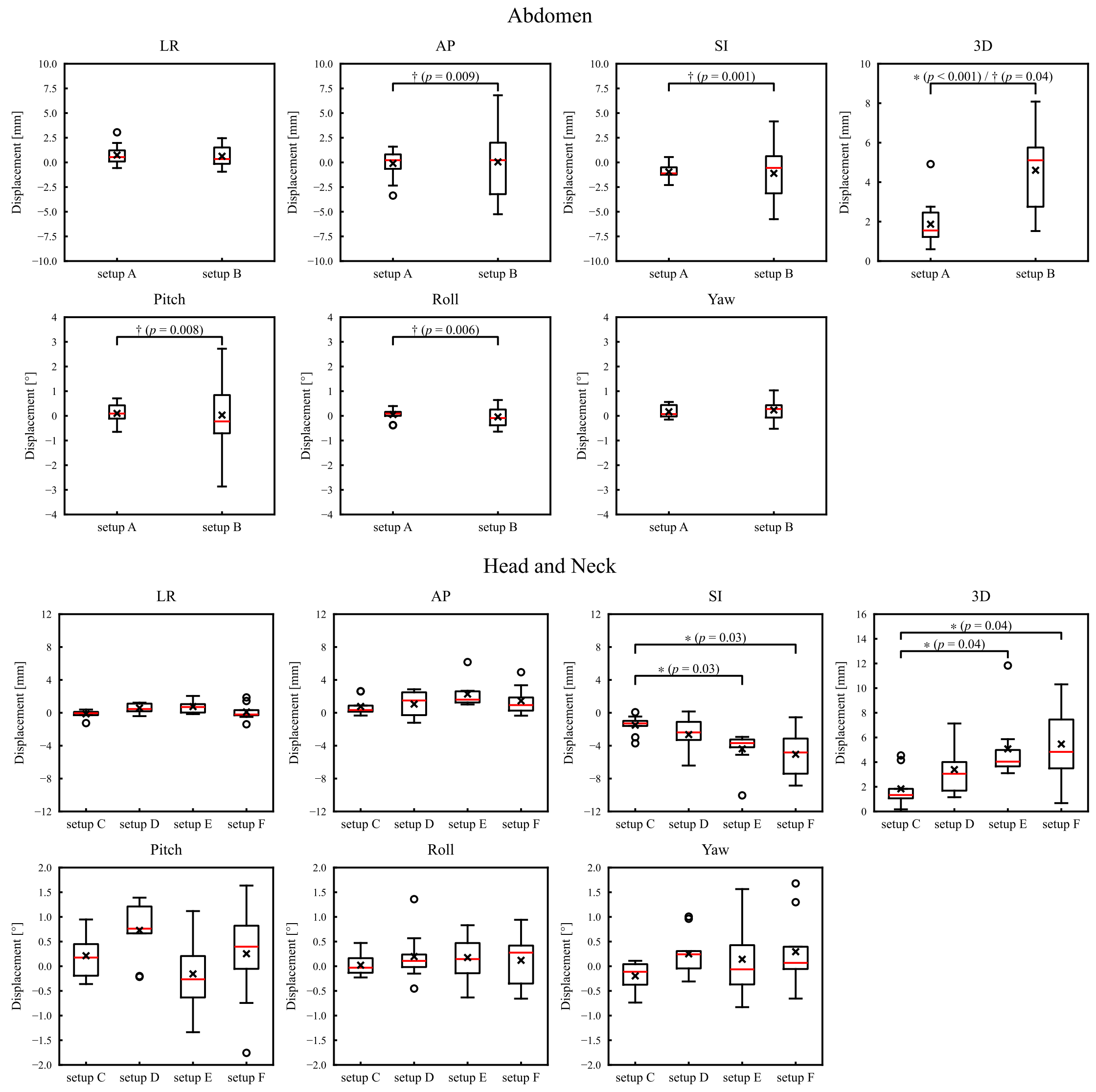

Figure S5. Comparison of the intra-fractional displacements at 20 min. Asterisk (∗) and dagger (†) represent a statistically significant difference in means and variances ($p < 0.05$), respectively. p values for the HN setups were adjusted with the Holm-Bonferroni method. Cross and circle marks represent mean values and outliers, respectively.
